# Additional Notes on Provincial Asylums for the Insane in France; with a Brief Notice of the Institution at Illnau, in the Grand Duchy of Baden

**Published:** 1852-01-01

**Authors:** John Webster

**Affiliations:** FELLOW OF THE ROYAL COLLEGE OF PHYSICIANS; CONSULTING PHYSICIAN TO ST. GEORGE AND ST. JAMES'S DISPENSARY, ETC.


### Original Communications.

### ADDITIONAL NOTES ON PROVINCIAL ASYLUMS FOR THE INSANE IN FRANCE; WITH A BRIEF NOTICE OF THE INSTITUTION AT ILLNAU, IN THE GRAND DUCHY OF BADEN.

BY JOHN WEBSTER, M.D., F.R.S., FELLOW OF THE ROYAL COLLEGE OF PHYSICIANS; CONSULTING PHYSICIAN TO ST. GEORGE AND ST. JAMES'S DISPENSARY, ETC.

THE complimentary criticisms contained in different medical periodicals, regarding my previous notes on French provincial asylums for the insane, which were published in the xii. and xiii. numbers of Dr. Winslow's "*Psychological Journal*," induced me again to go abroad during the recent autumn, in order to extend my observations respecting foreign lunatic institutions.

Accordingly, towards the end of last August, after resuming my portfolio and travelling portmanteau, I left London for the continent, with the special object of visiting various establishments of repute, in the north and north-eastern departments of France, seeing my former remarks only referred to those situated in the central and western provinces. Considering it now superfluous again to resume such subjects, after having already discussed, at considerable length, several points connected with the laws of lunacy, and the administration in French departmental asylums, I therefore refrain from investigating similar questions at present, but proceed at once to describe the institution first inspected, namely,—

### ARMENTIÈRES.

This asylum was formerly a religious institution, having been founded by an ecclesiastical society denominated "St. Jean de Dieu." For many years it has received insane patients within its walls, and is now appropriated for the treatment of male lunatics chiefly belonging to the department of the North. The institution is situated in the small manufacturing town of Armentières, about ten miles north-west of Lille, and not far from the railway leading to Calais. The buildings are rather old, and, although the adjacent gardens of the establishment are extensive, from being in the middle of a town having a population of at least 7000 inhabitants, the situation is not well chosen. Besides, as the portion occupied by inmates is surrounded by houses on three sides, whilst the main entrance and principal front form one side of the High-street, many of the objections inherent with position never can be corrected. Various alterations are, however, in progress within the interior; and, when the new wing is added to the building, which the authorities at present contemplate, some of the existing defects will be remedied to a certain extent, besides affording accommodation for upwards of 100 additional patients. This addition must prove a great improvement, and will relieve considerably the over-crowded state of the dormitories, which has become much augmented in consequence of various arrivals from Bicêtre; nearly 200 male lunatics having been recently transferred to Armentières from the former hospice, which was then overstocked with insane residents.

At the period of my visit, the number of lunatics in Armentières amounted to 496, all of whom were male patients, as the institution is exclusively appropriated for inmates of that sex; whilst females afflicted with mental disease are received into another departmental establishment, situated in the city of Lille. By this arrangement, the two sexes are kept entirely separate, and in distinct asylums maintained for their treatment. Amongst the 496 insane inmates now resident 40 were private patients, placed by relations or friends, who pay for their board and lodging sums varying from 400 to 1000 francs annually. In regard, however, to indigent lunatics, if they belong to the north department, the particular commune from whence the patient comes pays one franc per diem; but if the party is chargeable to any other department, one franc and twenty centimes are then demanded; whilst in the case of military patients, one franc and twenty-five centimes are allowed by the war minister for each individual. According to information kindly supplied at my request, the actual insane population of the asylum comprised 234 inmates considered wholly incurable, 75 laboured under paralysis, and 80 were classed as dirty patients; thereby leaving only 107 lunatics who exhibited any prospect of future convalescence. These statistical statements deserve notice, seeing they clearly indicate the serious nature of the maladies affecting a large proportion of the patients, if not the improbability of ever being able to effect any improvement in their mental condition.

The only official medical attendant of this extensive lunatic institution is Dr. Butin, a practitioner of much experience, and who has been attached to the establishment during many years. He resides in the asylum, but is not, as I understood, altogether debarred from private practice. There are no internes; consequently, when the physician is necessarily absent, it may happen that medical aid cannot be easily obtained at the moment required, which, in my estimation, is a defect in the organization of this, and of all similarly-officered establishments designed for the reception and treatment of lunatics.

When accompanying the physician during his morning visit to the different wards, the health of residents appeared, upon the whole, generally satisfactory. Several patients were, however, then in the infirmary, labouring under physical disease; one of whom was especially pointed out for my observation, being affected with tetanus of five days continuance. In this afflicted lunatic the symptoms seemed severe; he was wholly unable to open his mouth, and hence could only take liquids; the muscles of the neck were rigid, as also those of the body and limbs; indeed, the case appeared altogether of a most unfavourable description. Apparently, this severe malady was produced by exposure to cold during night-time, as the patient had been found lying under his bed quite naked, a few mornings before its commencement, when the temperature was low, whilst the lunatic also seemed at the time very far from being otherwise in robust health. At first, opium and camphor were prescribed, but apparently without benefit. Ether, which had been administered during the preceding twenty-four hours, apparently produced some alleviation of the symptoms; but, although slight, the effect seemed sufficient to encourage a continuance of that remedy; nevertheless, the ultimate termination remained very precarious; and, in reply to Dr. Butin, who politely asked my opinion, I feared the patient would not recover. After describing the history and treatment of this case, Dr. Butin also mentioned that he had met with other instances of the same malady—both traumatic and idiopathic—in the adjoining districts of France; which facts are remarkable, seeing this severe spasmodic disease prevails much less frequently in the cold and northern parts of Europe, than throughout warmer regions; for instance, in Italy, or even in the southern departments of this country.

Attaching great importance to the circumstance, whether personal coercion is usually employed, by the camisole or otherwise, at particular institutions, during the treatment of insane patients; and further, considering the extent to which such a system may be carried, always furnishes an instructive criterion of the principles actuating the professional authorities in attendance, I therefore, both here as elsewhere, made particular inquiries respecting the number of persons under any form of restraint at the period of my visit to any asylum. When making these investigations, I invariably endeavoured to obtain exact information regarding such an important subject; consequently, the various numbers recorded, in subsequent as in my previous notes, when discussing this question, do not exaggerate the amount of restraint employed in any institution, seeing only those cases which were personally observed are enumerated. Additional instances of the kind may, perhaps, have escaped my observation, which should have come within that category; but believing none existed, the various statements made throughout subsequent pages, in reference to the employment of mechanical coercion, ought therefore to be received as accurate.

The above preliminary observations respecting restraint in French lunatic asylums have now been made, to show that a constant desire prevailed, on my part, to be an impartial recorder of facts, whereby opinions enunciated, or the inferences other investigators might feel disposed to deduce, will be based upon authentic data obtained on the spot, and stated on my responsibility. Trusting these remarks may be kept in remembrance, when recurring to the subject of personal coercion in future pages, I would briefly observe:—On the morning of my visit to the Armentières institution, fifteen insane patients were confined by camisole, three of whom being likewise tied to the table at which they sat; one having, besides, a wire mask on his face, similar to the apparatus usually worn by fencers. The maniac thus accoutred was reported very violent, and often dangerous to other inmates; consequently, this machine was applied to prevent him inflicting injury either upon attendants or patients, as also from tearing his clothes, or eating objectionable substances.

During the year 1850, the movement of patients at this insane asylum was reported as follows;—admitted, 123; discharged cured, 29; died, 58.

Including the 464 inmates remaining under treatment on the 1st of January, 1850, with the 123 new cases admitted during the year, it was stated, in reply to my inquiries, that the aggregate 587 patients under treatment were classified into the five following divisions, according to the form of their particular mental maladies:—viz., 1—of mania, there were 228 cases; 2—dementia, 150 cases; 3—idiocy, 84 cases; 4—lypemania, 65 cases; and 5—monomania, 60 cases. But it should be added, that among the lunatics thus enumerated, 51 individuals were also reported as epileptics. From these statements, the reader may perceive, a large proportion of the patients at Armentières were either incurables, or offered very little prospect of subsequent recovery; which remark applies to many of the departmental asylums in France, as also to various county establishments for lunatics in England.

Cholera having occurred in numerous French asylums during the year 1849, when it prevailed epidemically on the continent and in London, I therefore considered it useful to mention, in my former notes, whether this disease had attacked the inmates of any of the institutions then visited. Influenced by similar reasons, I made inquiry respecting this point, during my more recent excursion, in order that the additional facts so collected might still further illustrate the progress of this severe malady, which sometimes prevailed like a pestilence. At Armentières, cholera proved exceedingly fatal during its invasion in 1849, when seventy-two patients lost their lives by that scourge, amongst a total mortality of 117 persons. Respecting the above visitation, it is also instructive to state, that forty of the seventy-two deaths took place during September; fifteen cases being reported in the first week, seven on the 8th of that month, four on the 9th, and seven on the 10th; whilst the remaining seven deaths by the same cause occurred at longer intervals. It thus appears, twenty-two deaths were reported during the first eight days of September; which fact becomes more worthy of notice, seeing the data now detailed exhibit a remarkable coincidence in respect of the period when the cholera prevailed in this asylum and at London, where it likewise proved fatal in a much higher ratio, during the first week of September, than at any other period of the entire season. It is, besides, important to remark, at the time this epidemic proved so destructive to the insane, it was equally lethal amongst the inhabitants of Armentières, where, during the season cholera prevailed, more than 500 persons are said to have died from that cause, exclusive of the seventy-two deaths reported amongst the lunatics. The malady seemed like a plague, and devastated the locality so much, that forty funerals were stated to have actually taken place in the town on one day. The fact of 500 deaths by the epidemic having occurred in a population of 7000 persons, shows the fourteenth part of the whole inhabitants were swept away by the disease in question; whilst the proportion of deaths in the asylum was double that of the town, or nearly one-sixth of all the lunatics then resident.

Although no farm is attached to this institution, the gardens adjoining afford means for employing some of the inmates in out-door work. In addition to such occupations, small gangs of patients, under the charge of attendants, are also permitted to labour in the fields belonging to townspeople; from whence they always return to dine in the asylum, but again resume work in the afternoon. For this employment, each patient receives a gratuity of 25 centimes per day, which is appropriated to form a fund for after benefits, or occasionally to augment their present comforts. Various inmates are likewise allowed to leave the asylum, under similar regulations, to work for persons in town, some as masons, and others to dig the foundations of new houses now in course of construction. This privilege is appreciated by the poor lunatics, and seems to be beneficial. Within doors, a few patients were, at the period of my visit, occupied in filling bobbins and other employments; but, speaking from personal observation, the labour system does not appear to be carried forward so zealously in this establishment, as I have witnessed elsewhere; and it seemed as if the means for employing patients were scanty, notwithstanding the desire no doubt existed.

All the patients were clothed alike, which system I consider defective in some respects, although rather common in French departmental asylums. Similarity of costume is too monotonous, and looks uninteresting; whilst a little variety, even in the colours of the materials employed as clothing, would prove advantageous by attracting the attention of inmates. But there is so much uniformity, military discipline, and similar outward formalities in the habits, or the ordinary occupations of every day life, amongst the inhabitants of France, that customs which may appear, to strangers from another country, influential in regard to the effects they produce, to natives seem often of so trifling importance as scarcely to merit observation.

The food supplied to the inmates was good, the bread used throughout the establishment being all of the same quality; which did not prevail some years ago, when the indigent patients received an inferior kind from the pensioners, whereby complaints on their part were frequent. These have now been entirely obviated, by distributing to every patient bread of the same description.

Although this asylum cannot be compared with many others in France, relative to the accommodation it affords to inmates, nevertheless, the recently constructed dormitories, and some of the old apartments, which have been improved by altering the interior, and making better ventilation, appeared well adapted for the purposes proposed; and as soon as the new wings now projected are finally constructed, the present conveniences will be farther extended. In consequence of the large number of patients under treatment, the wards were rather crowded, still, every effort is made to remedy the attendant inconveniences; and although many of the sleeping-rooms cannot be compared with the same kind of apartments in new asylums, they are, nevertheless, of a fair description. Formerly, the beds were all of wood; but these are now being replaced, throughout the entire establishment, by iron of a good and improved construction. The ancient cells for confining excited and dangerous maniacs, were horrible dens, but they are no longer occupied; the new apartments at present used for that purpose being much better constructed, although inferior, in many respects, to those I have seen elsewhere.

During my survey of this institution, the inmates of the various divisions appeared quiet, and conducted themselves in an orderly manner, when the physician made his morning visit. Whilst performing this important duty, all the patients able to attend were arranged in a line, under the trees of their respective court-yards, or in the open galleries which surround these enclosures. In this manner, every individual was seen and questioned without difficulty, or loss of time; besides which, the necessity of each patient keeping his proper position, and of exercising some self-control over his movements, during the physician's professional inspection, doubtless proved beneficial. Those inmates unable to attend this military-like examination, or who laboured under physical disease, being subsequently visited by Dr. Butin in their different dormitories, or at the infirmary.

Amongst the inmates pointed out to my notice, during this visit, one deserves mention, on account of being the individual whose case was described, and a representation of his features given, many years ago, by Esquirol, in that author's publication, "Des Maladies Mentales." The patient now alluded to is "Aba," described as an example of idiocy, at page 318 of the above work, whilst his portrait forms plate 22 of the accompanying atlas. As this case is well known to the profession, it hence appeared more interesting. Aba was formerly an inmate of Bicêtre, but he has for some time resided at Armentières; and although now an old man—quite idiotic—he still retains some resemblance to the original likeness given by Esquirol.

Several pellagrose lunatics were likewise observed in the asylum; thus showing that the above affection, so common in Italy, and also occasionally in some districts of France, is not unknown in the northern departments. Besides other patients deserving notice, one particularly attracted my attention from delivering three letters to Dr. Butin, to whom he made a profound salute on his approach, and after requesting they might be forwarded to their different destinations, he retired with another obeisance. This poor maniac believed he was the "Grand Seigneur," and often wrote letters to great personages, from imagining he had frequent important political relations with different European sovereigns. The missives now delivered were addressed, respectively, to the "Minister of War of the French *Emperial* republic," "to the Attorney-General of the *Emperial* republic," and the third had for superscription, "To the High Préfet of Dublin, in Ireland." Subsequently, I spoke to this crazy grand seigneur, who assumed an air of dignity, when he formally presented me with a piece of paper, the size of a calling-card, upon which were written his various assumed titles; and as he seemed satisfied with the reply made, that the several communications would be duly forwarded, we left him in apparent good humour.

Like many other asylums in France, the quantity of water supplied is defective at this institution, even for necessary purposes; at least, it would be so considered according to the opinions usually entertained by Englishmen, who very generally think so necessary an element, in all public establishments, especially receptacles for the insane, cannot be too abundant. In consequence of this deficiency, water-closets are unknown; whereby, the portable substitutes require removal every morning, which entails much additional labour on the attendants, besides being by no means salubrious. But custom familiarizes many objectionable practices; and as the system now alluded to is nearly universal, throughout all hospitals and asylums on the continent, it excites less attention than it would in England. However, this seems no argument in favour of present habits; on the contrary, both reason as well as health point in another direction, and indicate the necessity of constantly having a plentiful supply of so necessary an article as water in every public institution.

Although the managing authorities of the Armentières lunatic asylum are anxious to improve its capabilities, and to correct the various defects inherent to all buildings, not originally intended for the reception of insane patients; nevertheless, being situated in the middle of a town having a considerable population, with streets or houses on almost every side, whilst some of the court-yards are only prevented from being overlooked from neighbouring thoroughfares by dead walls, which impede the view, and the institution being only open on one side towards the country, where the gardens adjoin, few situations could be more objectionable. Instead of constructing additional wards, or of endeavouring to improve the present dormitories, it would be much more judicious, and certainly far better adapted than for the treatment of patients labouring under mental disease, were the destination of this establishment altered to a barrack, workhouse, or prison. In that case, a new and properly arranged institution might be constructed in a better locality, which would be worthy of so rich and important a department as that of the North, besides being conformable to modern advanced civilization.

### LILLE ASYLUM.

The building now known under the above designation was formerly a convent, which, at even a very early period, received lunatics within its walls; although, for some time past, the establishment has been exclusively appropriated for the reception of insane female patients. This institution is situated in the city of Lille, quite close to the new station of the Northern Railroad. Being surrounded by houses in every direction, and having very crowded streets on three sides, the situation is very noisy, and most inappropriate for an asylum, especially as the great thoroughfares leading to the railway pass under its windows. If the position was bad formerly, it has become much more objectionable, since the Paris and Lille railway has engrossed the chief traffic betwixt the French capital, England, Belgium, and the north of Europe. Indeed, a worse site for a madhouse could not be anywhere selected. Either the railway station must be closed, or the asylum removed to another locality. Which of these contingencies will happen, it is easy to foretell.

Internally, the arrangements are exceedingly defective; and it could not be otherwise, considering the period when the building was constructed, as also its original destination. The entire structure seems more adapted for a prison than a lunatic asylum; whilst the central court-yard, used by patients as an airing-ground, being of limited extent, and surrounded by high walls, or overlooked from the interior windows, is utterly incompatible with the purposes required in such an establishment, particularly with so numerous a population. Besides, as the space never can be enlarged, and any improvement appears nearly impossible, the case is almost hopeless.

As previously stated, none but female lunatics can be admitted into this asylum; and only those belonging to the north department are received as indigent patients. At the period of my visit, the total number of insane residents amounted to 335; of whom only 60 were classed as curable patients; whilst 5 were paralytics, and 17 epileptics; the remaining 253 being all incurables. Dr. Gosselet, a gentleman animated by great zeal for his profession, and of considerable attainments, is the only medical officer attached to this asylum, but he has neither assistants nor internes. In addition to this feature in the medical staff, owing to the limited accommodation within the institution, and its numerous inmates, the present physician resides at a private house in an adjoining street, which must consequently prove very inconvenient to all parties. However, as Dr. Gosselet gives his whole time and attention to professional duties at the establishment, whilst he frequently visits particular cases twice, or even three times a-day, when necessary, the inmates never remain without sufficient medical attendance.

During 1850, the following statement, kindly furnished, shows the movement of patients for that year:—Admitted, 84; discharged, cured, 16; died, 25. Amongst the deaths recorded, 10, or two-fifths, occurred in the months of January and December, being the cold season. Again, of the whole 25 fatal cases, 13 died from diseases of the brain or its appendages; 7 by pectoral affections; and 4 from abdominal complaints; whilst the remaining death was a suicide by strangulation. Unlike various public lunatic establishments in France, cholera did not attack any resident in the Lille asylum, either during 1832 or 1849; which facts become more remarkable, seeing that the epidemic prevailed in this district, especially at Armentières, as mentioned in a previous paragraph.

Notwithstanding the inmates seen in most of the dormitories were rather tranquil throughout, particularly if contrasted with female lunatics usually resident in French asylums; nevertheless, those occupying the agitated division were very noisy and excited. Amongst the 335 insane patients under treatment, 28 individuals were restrained by camisoles, on the morning of my visit to the institution; of whom, 25 were actually congregated together in one apartment, all being the worst and most violent cases in the establishment. Many of these afflicted human beings were likewise strapped to the seats they occupied, in order to prevent contact with others in the vicinity; whilst some were likewise attached by cords to the walls of the room, behind the wooden benches they sat upon; and further, one woman had her feet also tied together. The noise, screaming, and agitation of so many furious female maniacs, all confined in the same, but not very spacious apartment, were really harrowing to the feelings, and certainly, the visit paid to this division constituted one of the most painful spectacles I have ever witnessed.

In extenuation of the numerous examples of personal coercion, now recorded at the Lille asylum, it should be mentioned that, the strait-waistcoat is at present much less frequently employed than formerly. For instance, about three years ago, when Dr. Gosselet was first appointed physician to this institution, the average number of persons usually in camisoles ranged about sixty per day; although the aggregate amount of resident lunatics was then less than at present. Notwithstanding every effort is now made, both by the physician and attendants, to avoid restraint, it becomes often very difficult to eradicate a bad system once established; especially if supported by usage, and in so confined a locality as the Lille asylum. However, the number of cases in camisoles is occasionally less than the amount now reported; in proof of which, it was stated that, on the day previous to my visit, the total patients so coerced were only sixteen throughout the entire establishment. Still, the above ratio is enormous; and therefore, Dr. Gosselet must not relax in his efforts to extend the non-restraint system, whenever possible.

Formerly, a great many cells, or more correctly speaking, dungeons, were in constant use, at this asylum, for confining dangerous or refractory maniacs. Most of these symbols of a barbarous age are at present entirely closed, or converted to other purposes; with the exception of nine, which are still occasionally occupied, when the seclusion of a patient is considered advisable. But these apartments are now in a very different condition, compared with ancient arrangements. The prison-like iron bars on the windows have been removed; and instead of the antiquated wooden beds, like cages, into which an excited lunatic was sometimes thrown, and there kept, as wild animals often are in a menagerie, modern iron bedsteads of an excellent construction have been substituted. In short, the existing cells are merely small rooms, each for the reception of one patient. It should be likewise stated that the above apartments adjoin the dormitories, and are quite different from those observed at Bon Sauveur, described in my former notes. Further, many of the old cells have been taken down, and made into large sleeping rooms, in order to augment the available accommodation; as there are always a number of applicants waiting for admission, although the asylum is even now overcrowded with inmates.

Constant endeavours are made by the physician, and director, M. De Lussatz, —both most zealous in the cause—to employ the lunatics in some kind of occupation, wherever practicable. In one department, I saw upwards of 130 insane females engaged in various sorts of work, under the superintendence of several sisters of charity, besides attendants, who formed, altogether, a gratifying spectacle. Some of the inmates were knitting, others sewing, and several were making clothes for the household; whilst, about twenty were engaged in lace making, which constitutes the ordinary occupation of many females in this portion of France. One woman amongst the group was specially pointed out for observation, as she then plied her bobbins with great zeal in weaving a most beautiful piece of lace, which was, apparently, as well made and cleanly preserved, as if it had been the work of a first rate perfectly sane artizan. Speaking generally, nearly two-thirds of the residents of this asylum were occupied in some kind of employment; either in the manner just described, in household work, cleaning the apartments, making or repairing clothes, cooking, and in whatever the authorities considered advantageous, or likely to engage attention. This zeal in promoting labour ought to be taken into consideration, when judging the institution as a whole; hence, impartial visitors should not solely look upon the number of camisoles employed, but upon other features likewise.

Subsequently, I visited the refectory, whilst 140 inmates were at breakfast. After grace was said by the presiding sister of charity, and an appropriate response from the audience, the party all sat down without confusion, and in messes of ten at each table, where they eat their meal with much quietude. Each division was served by one of their own number—also a patient —who acted as superintendent, and became responsible that all were attended to properly. This official wore a shawl of a particular pattern, in order to mark her position, and to show she had delegated authority over other patients. Such an arrangement appeared judicious; and produced beneficial effects, not only upon the parties so distinguished from the rest, but it also acted as an encouragement for others, to obtain the more elegant shawl, thus exclusively worn by their insane companion-attendants. In another eating-apartment, although the party was not so orderly in appearance as the preceding, about fifty agitated female patients were at breakfast, and arranged in nearly a similar manner to the former. These unfortunate victims of insanity certainly made more noise than the previous assembly; but they sat together pretty regularly, and endeavoured to restrain themselves during the repast they were partaking. This was satisfactory; for if able to accomplish such results, one step is gained in the management of lunatics.

Amongst the group collected in this apartment, one insane female was pointed out, who had resided many years in the asylum. She had been formerly very agitated, if not dangerous; and, therefore, the attendants frequently put her under restraint, even for some continuance. Through a change of system adopted towards this excited maniac, she became quite different in many respects, and behaved much more orderly. Previous to recent improvements, and the alterations made in the general management of this institution, the inmate now referred to actually remained, during many years, shut up in one of the ancient cells then used for confining furious patients, where she often lay entirely naked, slept generally on straw, and otherwise conducted herself in such a noisy manner, as apparently to justify the treatment pursued. After the lapse of years, this afflicted female became gradually tranquil, and ultimately showed very marked improvement. At present, she dresses herself in the morning, joins other companions at the work-table, sleeps in a dormitory with several patients, and behaves almost like a rational creature, being quite different from the wild-looking person she formerly appeared. Such highly gratifying results having been produced by kindness, and the improved moral treatment adopted in this, as in other examples, constitute strong arguments in favour of its extended application.

Notwithstanding most inmates of the agitated divisions, particularly those confined in camisoles, were often very boisterous, still, with such exceptions, the general aspect of the institution was, upon the whole, tranquil, especially for female maniacs. In the work-rooms this feature was chiefly remarked; which proves incontrovertibly the great advantages generally produced by giving employment to all lunatics, both males and females, the latter of whom are usually more agitated and noisy than the other sex. This is the case in France; at least, according to my individual observation.

One important characteristic in the treatment of patients, at this asylum, deserves special mention in these pages—viz., the system of mutual instruction recently introduced. By this method, the authorities not only instruct ignorant maniacs in reading, writing, figures, and keeping accounts, which frequently the patient never knew before, but attention being thereby induced, dormant faculties even become so awakened as to produce beneficial consequences. Besides the good effects upon individuals thus engaged, as more advanced lunatics often act in the capacity of monitors, they also derive benefit by such occupations. Many become improved; some by teaching, others by learning; so that the condition of all parties is thereby ameliorated. Dr. Gosselet spoke highly in favour of the plan he had adopted; and, in my opinion, with much reason.

The provisions supplied seemed of good quality, and animal food was allowed five times a week to the patients. Their bodily health appeared generally satisfactory, and few inmates occupied the infirmary. These are gratifying facts, and show the care as well as constant attention paid, by the various officers of the establishment, to the unfortunate victims of insanity committed to their superintendence. Coinciding with Dr. Gosselet in many of the opinions he entertained respecting the treament of lunatic patients, I feel assured, had he more conveniences in the asylum, and were it less crowded by inmates, at the same time, had he all the appliances which many newly-constructed institutions possess, personal restraint would be much seldomer employed than at present. Considering the irremediable defects and former condition of such an old building, with the prospect of future improvements in its management, the authorities deserve credit for the ameliorations already accomplished.

Amongst the 335 insane residents now under treatment in the Lille asylum, 56 are pensioners, who pay from 400 to 1000 francs per annum. The accommodation for private patients is pretty good; but here, as in other divisions, a great want of space prevails; so that the apartments occupied by this class were, like the others, too crowded. Parties of pensioners are permitted to go out of doors to enjoy a promenade in the neighbourhood. Occasionally, indigent patients are likewise allowed similar indulgences, when the physician thinks such proceedings advisable. Great circumspection is, however, necessary in authorizing similar excursions, in consequence of the numerous wet ditches and canals connected with the city and its extensive fortifications. That such is no imaginary danger, appears by an occurrence which took place, soon after I left Lille. On this occasion, several lunatics—inmates of another establishment—having been conducted into the neighbouring fields, one of the party, notwithstanding the attendant's vigilance, jumped into the canal, near which they then were walking, and was drowned.

In this asylum, as elsewhere, every patient who dies is pathologically examined after death, in order to ascertain the diseased changes of structure which have supervened. According to Dr. Gosselet's observation, some disorganization of the brain or appendages were invariably found, more or less appreciable, but always sufficient to show, that mental affections attacking lunatics are the consequence of organic disease. On these points his evidence seemed conclusive. Respecting another subject, that physician likewise made some valuable observations—namely, the evident influence which certain atmospheric conditions often exert upon mental maladies. Thus, insane patients seemed less excited during rainy, than in dry weather. The barometric pressure of the air also produced apparent effects; whilst cold always proved injurious. According to the same authority, the pulse of lunatics usually becomes accelerated; not feverish or inflammatory, but quick and excited. Further, Dr. Gosselet decidedly said, all lunatics should be well fed, warmly clad, and uniformly treated as if sane, wherever practicable, and they ought never to be deceived by false promises, always kindly treated, and managed with firmness yet good temper; whilst insane patients should be induced to work through persuasion rather than by force, but certainly never by menaces of punishment.

Believing it wholly impracticable to remedy the existing defects in the Lille asylum—which must, on the contrary, augment every year—it hence becomes even more imperative than in the case of Armentières, to construct a new institution in a more eligible locality. For so rich a department as the North, there ought to be no hesitation in following the good examples set by various public bodies of the country, such as the Lower Seine; where the present asylum of Saint Yon will be appropriated to female lunatics, when the new institution near Rouen for male patients is finished. The council-general of the north department will, I hope, receive the remarks now made by a stranger in the spirit intended, as they originate from a sincere desire to improve the condition of those helpless lunatics now confined in the present building; which, even had it been originally better constructed, is very different from the accommodation provided in many districts of France. To such a determination the government must at last come; and the sooner an alteration is effected, so much the more beneficial to inmates, and higher honour will then be acquired by departmental authorities.

### ASYLUM AT CLERMONT.

This establishment is the private property of the Messrs. Labitte, and was founded about twenty years ago by the father of three brothers, who now superintend its different departments; one being director, another chief physician, whilst a third manages the agricultural operations of a large farm belonging to the institution. The buildings and gardens constituting the asylum, properly speaking, are situated on a declivity, inclining towards the south-east, close to the town of Clermont, built upon a hill in the department of the Oise, which is remarkable for its magnificent terrace surrounding the ancient castle, and from whence the spectator possesses an extensive yet beautiful view of the surrounding country. The distance from Paris is about fifty-two miles; and being near the railway betwixt that city and Amiens, the situation is exceedingly convenient for travellers, or persons having business at the establishment.

Although a private asylum, indigent insane patients are admitted, in accordance with an agreement entered into by the public authorities of four departments—viz., the Oise, the Seine and Oise, Seine and Marne, and the Somme. Besides the above class of lunatics, pensioners belonging to other grades of society are likewise received, who pay from 500 to 1200 francs per annum, for their treatment and maintenance at this institution. The court-yards are numerous, well arranged, and entirely separate; whilst the ground upon which the dormitories are constructed being extensive, there is no want of space. The different divisions seemed well laid out, were open, and airy; and, the residences for patients being usually two stories high, they supply appropriate yet ample accommodation. Besides which, great order with cleanliness appeared manifest everywhere. Dr. Labitte, as already stated, is the attending physician; he has also one assistant, an interne, and a pharmacien—all being resident on the premises.

At the period of my visit to Clermont, the number of lunatics under treatment amounted to 876 inmates, consisting of 390 male and 486 female patients; consequently, this asylum is one of the largest throughout France. Amongst the present residents 68 were pensioners, 57 being men and 11 women. In regard to the various forms of mental disease affecting this large population, the following facts, kindly supplied by Dr. Labitte, must be interesting to readers, as they indicate the actual condition of present inmates. Thus, 101 were classed as epileptic patients, 36 being males and 65 females; those labouring under general paralysis amounted to 37, comprising 31 men and only 6 women; whilst 74 were dirty patients, consisting of 27 male and so many as 47 female lunatics. Again, it should be mentioned as an equally important part of statistics that, not less than 682 patients now resident were considered incurable, 249 being males and 433 females; thus leaving only 194 lunatics who exhibited any prospect of future amendment.

Respecting the actual amount of restraint at the period I inspected this extensive establishment, according to notes taken at the time, it appears 14 females were in camisoles, some being also tied to their chairs to prevent their falling, and so causing injury; whilst, in the male divisions, four individuals had also strait-waistcoats; but all were otherwise free. Nearly the whole of the above number were indigent patients; only one male and one female pensioner being comprised, amongst the eighteen inmates under coercion. Notwithstanding the statements now made, it ought to be distinctly understood, in justice to Dr. Labitte, that he is an opponent to the employment of camisoles in treating maniacs; and only has recourse to such physical measures, to prevent furious lunatics injuring others or themselves. In proof of this feeling, a male patient was shown, who had been confined to his chair by a camisole, in another lunatic asylum, during eighteen years consecutively, on account of being very violent and considered dangerous. After his admission into the Clermont establishment, the restraint having been soon removed, he was put to some occupation. In a short time this lunatic became quiet, conducted himself as an industrious orderly workman, and was so much improved by this mode of proceeding, that the camisole has never since been applied; thus illustrating most conclusively, the great benefits of non-restraint.

Besides other illustrations of a somewhat analogous description, it ought also to be mentioned that many of the cells, originally constructed for confining excited patients, have been abolished, and new dormitories constructed, in addition to other improvements now in progress. At present, there are twelve cells for male, and fourteen similar apartments for female patients, wherein they are placed when seclusion is thought advisable. The iron bars formerly seen in many windows have been removed, in order to substitute wooden shutters; and every effort is making to obliterate any prison-like appearance, throughout the various buildings.

During my perambulations through this extensive institution, great regularity respecting the details necessary, in so large a population, seemed to be established. The attendants appeared active, and keep an accurate register of the various employments in which patients are occupied, for the physician's information. Further, if any event had occurred since his previous visit, or it was necessary to direct attention to new matters, the facts having been written on a slip of paper, this report was always handed to Dr. Labitte, on entering the particular division to which it referred, so that nothing might be overlooked.

During the year 1850 the following return, taken from the official register, indicates the movement of patients at this asylum:—

| | Males. | | Females. | | Total. |
|---|---|---|---|---|---|
| Admitted . . . . . . . . | 132 | ... | 134 | ... | 266 |
| Discharged Cured . . . | 20 | ... | 33 | ... | 53 |
| Died . . . . . . . . | 65 | ... | 85 | ... | 150 |

Amongst the 65 deaths of male patients now reported, 50, or about seven-ninths died from disease of the brain or nervous system; whilst three-fourths of the deaths in female patients exhibited, as the apparent cause of dissolution, affections of the abdominal viscera or pelvic organs. These are interesting facts, and have now been recorded in order to ascertain whether other pathologists have made similar observations.

Believing it will prove instructive to give several details, illustrating the movement of inmates at this large insane establishment, during a series of years, in order to compare the ratio of cures and deaths with the total admissions, it may be mentioned that, from the 1st of January, 1840, to the 31st of December, 1850—being a period of eleven years—2346 insane patients of all classes were admitted; the number discharged cured being 504, whilst 1114 died. Amongst the latter, it should, however, be added, 118 deaths arose from cholera. Taking the figures as now stated, only 21.48 per cent. of the patients left the asylum convalescent; whereas not less than 47.06 per cent. died, calculated according to the admissions. Excluding the 118 deaths by cholera, which occurred in 1849, as, in some degree, exceptional cases, the mortality, nevertheless, amounts to 42·02 per hundred. This makes a large ratio, and leads to the conclusion, that mental maladies usually affecting lunatics received from neighbouring districts into the Clermont institution, are often of a very inveterate or incurable nature.

When cholera prevailed throughout so many asylums in France, during 1849, nowhere perhaps, unless at the Salpêtrière in Paris, did this epidemic malady produce such ravages, as in the lunatic institution under discussion, where 118 patients, comprising 28 men and 90 women, became victims, besides 9 attendants of the establishment. And as the total cases recorded amounted to 216, it hence appears, nearly 59 per cent. of the persons attacked died by that disease. Some time previous to its appearance in this asylum, the epidemic had prevailed at Clermont; however, when cholera actually supervened, very few examples remained amongst the 3000 inhabitants of that town. Another fact also deserves notice; viz., no case was reported from a large central prison for females, which almost adjoins the institution on the north-west, notwithstanding it then contained numerous prisoners. Previous to the 26th of June, the asylum remained perfectly free from cholera; but from that date to the 31st of July, or a period of five weeks, during which it existed, all the 127 deaths were reported, patients and attendants included. Afterwards, the malady ceased entirely. In addition to these statements respecting the recent epidemic, it is also instructive to mention that, out of 122 cases carefully noted at the time, 86 commenced during the night; whereas, only 36 attacks began from 11 A.M. to the same hour in the evening. The disease likewise affected fewer male than female patients; and, besides, proved to the former much less destructive; seeing 147 cases occurred amongst 535 female lunatics resident; whilst, out of 340 inmates, only 46 men were similarly affected. According to these data, 13.52 per hundred, amongst male patients, were attacked by cholera, of whom 60.08 per cent. died; whereas, the ratio was 27.45 per hundred, of insane females affected, which gave a larger proportion of deaths, or 61.22 per cent., many being old and infirm people. Residents labouring under dementia and epilepsy, especially males, more frequently suffered from cholera than curable patients; with the exception, however, of epileptic females, who were less numerous, in comparison with other forms of mental disease.

Judging from outward appearances, the general health of most residents seemed good; and as very few patients were under treatment in the infirmary, the physical condition of so large a population appeared satisfactory; being quite different from the autumn of 1849, as previously reported. Having large gardens adjoining the asylum, extending, with the various court-yards, to 37 acres, ample means are thus provided for employing insane patients in horticultural pursuits. Besides such opportunities for out-door work, as the farm attached to the asylum contains 247 acres—the most extensive establishment of the kind belonging to any insane institution in France—there is always plenty of agricultural employment at command. Indeed, sometimes as many as one hundred lunatics are engaged in the fields, both morning and evening. Some also work in the smithy, bake-house, and carpenters' shops; whilst others are engaged as tailors, shoemakers, and so forth. The females being also occupied in making or mending clothes, in the kitchen, washing-house, or at different kinds of household work. In short, to give employment to the insane residents is constantly kept in view at this institution; in which, about one half of the lunatic population are usually occupied, whereby their bodily health and mental afflictions are ameliorated.

Reviewing all the facts recorded during my visit to the Clermont asylum, I can justly say, the proprietors deserve commendation; whilst its management does credit to the three brothers who conduct so extensive an establishment; which, in many respects, including pecuniary considerations, becomes a very responsible undertaking. Although situated quite close to the town, nevertheless, the buildings being altogether disconnected with any adjacent houses, and not overlooked by neighbours, the inmates are never disturbed, nor can others be annoyed by their presence. In addition to possessing an extensive view of the surrounding country, its locality is considered salubrious, notwithstanding the ground floors of several dormitories are said to be occasionally damp, especially after heavy showers, in consequence of the moisture then collected from the upper garden and court-yards. The supply of water, as in other asylums, is rather defective, but not in comparison with many similar establishments.

Previous to taking leave of Dr. Labitte, for whose courtesy my best thanks are due, we entered the chapel belonging to the institution, where a large number of patients and attendants had assembled. Both male and female lunatics were occupied in their customary devotions, and behaved tranquilly during service, like any ordinary congregation. The men being ranged on one side, whilst the women occupied the opposite; and, considering most of the audience were insane, the scene thus displayed was highly interesting. Looking at the decorous behaviour, and cleanly appearance of the persons present, as also the general quietude everywhere prevalent, with the gay caps, red shawls, and blue gowns of the women, relieved by the black dresses or snow-white hoods of sisters of charity, and various men attired in blouses, the whole spectacle appeared exceedingly picturesque. After contemplating, during a short period, this numerous assemblage of human beings, deprived of reason, yet contented and apparently sane, I quitted Clermont having experienced many pleasing impressions.

### CHÂLONS ASYLUM.

This public institution adjoins the town whose name it bears, known as the capital of the Marne department, and also famous in history on account of the great defeat which Attila and his Huns sustained, A.D. 431, close to its ancient precincts. The situation occupied by the asylum is open, airy, on rather elevated ground, near the suburbs, and is evidently well chosen. The house was formerly a mendicity depôt, but many additions having been made, it then became a receptacle for lunatics, and has been so occupied about thirteen years. Extensive alterations are still in progress, whilst new dormitories have been recently added, with cells of an improved construction for the seclusion of agitated patients. These apartments are spacious and convenient, especially the dormitories, which seemed the best of the kind I have met with any where. The court-yards and gardens, although not extensive, are judiciously arranged; whilst the work-rooms being cheerful, well lighted, and judiciously ventilated, appeared highly creditable to the architect and superintending authorities.

The Châlons asylum only admits indigent patients belonging to the department of the Marne, which every person knows forms part of the province of Champagne. Indeed, amongst the curiosities of this district, usually visited by travellers, the celebrated wine cellars of Messrs. Jaqueson and Co. deserve notice, as they extend for miles, and contain, on an average, about three million bottles of his much prized sparkling beverage. So extensive are the buildings in which this champagne is *manufactured*, that report asserts the straw and wood sheds, with those used for storing casks, were taken by Government, during six months, as a barrack, to lodge 4000 men; whilst the open courtyards served as a place to perform their military manœuvres. Being constructed near Châlons, on a moderately high hill, the various houses look like a small town, and form a prominent object in the distance.

Dr. Giraud, very favourably known to the profession by his works on insanity, is the physician, and only resident medical officer of the institution. He is likewise director, but has no interne. There is, however, an assistant physician, who resides in Chlâons, and visits the asylum professionally when his attendance is required, or if Dr. Giraud happens to be absent through any cause. On the day of my visit to the establishment, the number of lunatic inmates amounted to 309; of whom 145 were male, and 164 female patients. About three-fourths were considered incurable; whilst four men were affected with paralysis, but not one case existed among the other sex. The bodily health of most patients was satisfactory, and very few cases were noticed in the infirmary; hence, judging from appearances, the general population seemed healthy, and the locality salubrious.

Respecting restraint, it is gratifying to report that no male patient was in any manner confined; but, at the period of my visit to the asylum, four female lunatics were in camisoles. Nevertheless, both in the male and female divisions, the inmates were orderly and quiet; indeed, it may be added, the women seemed less noisy and excited than has been sometimes noticed elsewhere.

Notwithstanding indigent persons are only admitted into the Châlons asylum, who belong to the department of the Marne, private patients are received from any other district; the payment, in these cases, varying from 400 to 1500 francs per annum, according to the accommodation. At the period these notes were written, the number of lunatics so classified was 63, of whom 29 were males and 34 females. The apartments for pensioners seemed good, and even some were of a superior description, which will be farther improved, when several proposed alterations are completed; and the various additions now contemplated cannot but prove most useful, seeing the proportion of insane persons in this department is rather higher than in many other districts of France; the ratio being, according to recent investigations, one lunatic in every 1155 inhabitants.

During the year 1850, the movement of patients at the Châlons asylum was reported as follows:—

| | Males. | Females. | Total. |
|---|---|---|---|
| Admitted | 37 | 36 | 73 |
| Discharged cured | 30 | 15 | 45 |
| Died | 13 | 8 | 21 |

Amongst the deaths recorded, a large proportion, according to the autopsies subsequently performed, were apparently produced by cerebral disorganization.

Dissimilar to various other French institutions, no death from cholera occurred at the Châlons asylum during 1849; notwithstanding that epidemic malady prevailed in the town with some severity, and several fatal cases even took place amongst the residents of houses not far from the public entrance to the establishment.

Much attention is constantly paid to employing patients in various kinds of occupations. Upon an average, about two-thirds—a high proportion—are usually occupied, whilst 114 insane inmates actually received recompences and gratuities for different pieces of work they had performed during last August. This system of making an allowance to lunatics for labour accomplished, proves a great encouragement to the poor recipients, as it augments their present comforts, besides often making a small fund for future necessities.

No farm being attached to this asylum, out-door work is chiefly obtained in the adjoining and well arranged gardens, in which I saw various lunatics busily occupied. Gangs of inmates, accompanied by their attendants, are likewise permitted to labour in the fields of neighbouring farmers, from whence they return in the evening, as if all were reasonable creatures. This plan is considered highly beneficial, and deserves being mentioned in these pages, as an example for imitation by other establishments. Besides the above employments, every kind of work necessary in the domestic economy and management of such an institution is performed by inmates. Numbers, especially of the female patients, were busy in their respective work-rooms; some in making and mending clothes, or in preparing linen, besides washing, cooking, cleaning apartments, and so forth; indeed, the general aspect of the asylum looked more like a factory than a mad-house; whilst great tranquillity prevailed throughout the entire building. I stated these impressions to Dr. Giraud at the time, and added that the establishment over which he presided was one of the quietest I had hitherto inspected. Compared with its former condition, great ameliorations have been accomplished in the Châlons asylum; whilst the proportion of fatal cases recorded have lately, and almost annually, decreased. Thus, in 1840, the ratio was one death in seven patients, which gradually fell to only one in seventeen during 1849. This decided alteration became more remarkable after recent improvements were instituted, but especially subsequent to the appointment of a resident physician.

Amongst the patients now under treatment in this departmental institution, one was pointed out, who deserves some notice in the present report, seeing he was not only the oldest inhabitant, but otherwise also interesting, from having served as an officer in the army of Italy, when commanded by Napoleon, and under whom he fought at the battle of Marengo. This veteran soldier of the empire had attained his ninety-second year, and, judging from outward appearance, may yet survive some time longer, and so constitute perhaps one of the last links connecting the former with the present republic. Nay more, having formerly served the first emperor, perhaps the old lieutenant may yet become the subject of a second imperial sovereign, sprung from the same stock, and governing the country.

Another insane resident of a different description, but viewed professionally, more instructive, and respecting whose capabilities I can speak with confidence, notwithstanding his intellectual deficiencies, also came under observation. The party now alluded to was clerk in the director's office, and superintended many details in that department. This poor fellow is very useful as an amanuensis, and labours with his pen like any sane person. Indeed, most of the official statements contained in previous pages were obtained through his instrumentality, all being, of course, subsequently submitted to Dr. Giraud, in order to insure accuracy; since any statistical information, of whatever kind, without such an essential qualification, becomes utterly worthless.

The inmates have animal food five times a week, whilst the other articles used seemed of good quality, and the culinary apparatus, as in most French asylums, appeared in excellent order; whilst the commissariat department was both efficient and properly regulated. Although the various substances which constitute the ordinary dietary of insane parties in English asylums are often of a better quality than throughout France, nevertheless, in the latter country, they certainly understand the mode of cooking, especially for large institutions, much better, and even more economically than on the English side of *la*

*Manche.* Considering the judicious feeding of lunatics is a most important point in their treatment, I would remark that the *cuisine* at this asylum attracted special attention; and although by no means an advocate of meagre or fast days for insane patients who require, on the contrary, to be better and more nutritiously fed than even sane persons, still the soup and vegetables supplied in French asylums, on Fridays and Saturdays, are generally properly prepared, whilst the bread supplied is abundant, and usually of excellent quality.

Being nowhere overlooked, notwithstanding its vicinity to Châlons, this departmental asylum is well adapted for the reception of insane patients. When the new dormitories are finished, and the improved cellular apartments, for secluding excited or dangerous lunatics, are completed, both may be taken as good models for imitation. Having two opposite entrances, attendants can at all times gain access to a cell, even although the occupant may place his body against one of the doors, in order to prevent free egress, which sometimes happened in those constructed upon the old principle, and so proved dangerous. In the new buildings such contingencies will be obviated; and in taking leave of the Châlons asylum, I would again remark, these apartments, as also the dormitories, are admirable structures.

(*To be continued.*)

